# Personalia

**Published:** 1914-04-25

**Authors:** 


### Personalia.

Dr. F. M. Sandwith, senior physician at the Albert Dock Hospital, has left England with Mr. Wickliffe Rose, of the Rockefeller Institute, who is engaged on an expedition in connection with ankylostomiasis. This inquiry into the various forms of hookworm disease will occupy some time. Dr. Sandwith is associated with the work in regard to this subject in Egypt and Ceylon. He is expected back in London in June.

